# Fatal Invasive Mold Infections after Transplantation of Organs Recovered from Drowned Donors, United States, 2011–2021

**DOI:** 10.3201/eid2907.230524

**Published:** 2023-07

**Authors:** Karen Wu, Pallavi Annambhotla, Rebecca J. Free, Jana M. Ritter, Brooke Leitgeb, Brendan R. Jackson, Mitsuru Toda, Sridhar V. Basavaraju, Jeremy A.W. Gold

**Affiliations:** Centers for Disease Control and Prevention, Atlanta, Georgia, USA

**Keywords:** mold, donor-derived infection, solid organ transplantation, fungi, *Scedosporium*, mucormycosis, invasive mold infection, drowning, United States

## Abstract

Drowned organ donors can be exposed to environmental molds through the aspiration of water; transplantation of exposed organs can cause invasive mold infections in recipients. We describe 4 rapidly fatal cases of potentially donor-derived invasive mold infections in the United States, highlighting the importance of maintaining clinical suspicion for these infections in transplant recipients.

During 2011–2021, a total of 4,136 organs from 1,272 drowned donors were transplanted in the United States (*1*). Aspiration of water during drowning events can expose victims to environmental molds (e.g., Mucorales and *Scedosporium*) that can cause invasive mold infections (IMIs) in transplant recipients (*2*–*5*).

Solid organ transplant (SOT) recipients are at increased risk for IMIs because of predisposing factors that weaken the immune system (e.g., underlying conditions and immunosuppressive medications taken to prevent organ rejection) (*6*). SOT recipients might acquire IMIs upon reactivation of a dormant infection, progression from colonization to invasive disease, inhalation of environmental molds, or exposure to mold in the transplanted organ (*7*). Previous case reports of donor-derived IMIs involving drowned donors highlight challenges in investigating IMIs in transplant recipients (e.g., ruling out alternative exposures) and high death rates (*4*,*5*,*8*). To inform clinical practice and policies to improve patient outcomes, we describe the donor and recipient characteristics and clinical progression of IMIs determined to be potentially transmitted from drowned donors in the United States during 2011–2021.

## The Study

In the United States, suspected transplant-transmitted infections are reported by organ procurement organizations or transplant centers to the Organ Procurement and Transplantation Network for investigation by the Disease Transmission Advisory Committee (DTAC). As a member of DTAC, the Centers for Disease Control and Prevention (CDC) investigates select potential donor-derived IMIs to determine whether transmission occurred through transplantation and prevent further transmission. For this study, we included investigations involving a drowned donor and >1 recipient with a posttransplant IMI in which the donated organs were determined to be the potential route of transmission (*9*).

During 2011–2021, CDC conducted 3 investigations involving 3 donors who drowned and 9 SOT recipients who received organs from those donors. Transmission of an IMI potentially occurred to >1 recipient of each donor. We describe these investigations and related findings; information about donors’ time spent immersed underwater was unavailable.

For case 1, the donor was a 2-year-old boy who was found unconscious in a pool. Organ procurement occurred ≈6 days after hospital admission. An autopsy revealed congested lung parenchyma consistent with drowning and no findings consistent with IMI; a histopathologic examination was not performed. The recipient of both kidneys (index recipient) was a 23-year-old man with end-stage renal disease caused by lupus nephritis. He experienced graft thromboses after transplantation requiring transplanted kidney removal 8 days posttransplant; histopathologic examination revealed severe mucormycotic emboli. He died 10 days after transplantation. Autopsy revealed massive pulmonary emboli and severe necrosis in the abdominal and pelvic cavities. The recipient of the liver was a 2-year-old girl with congenital biliary atresia. After transplantation, clinicians noted decreased hepatic artery flow. The patient subsequently underwent transplant hepatectomy 3 days posttransplant. Histopathologic examination revealed thrombosis of multiple vessels, extensive hepatic necrosis, and angioinvasive fungal elements suggestive of mucormycosis. The patient died 4 days after transplantation. No other organs were transplanted.

For case 2, the donor was a 30-year-old man who had cardiac arrest while swimming. Organ recovery occurred 8 days after hospital admission. Autopsy revealed no gross or histopathologic evidence of IMI. The recipient of the liver (index recipient) was a 60-year-old man with cirrhosis caused by hepatitis B and C and hepatocellular carcinoma. Two days after transplantation, clinicians performed a posttransplant liver biopsy, which found no evidence of acute rejection or fungal organisms. Three days later, the patient had worsening hepatic and renal function. Emergency laparotomy revealed liver necrosis with absence of blood flow in the portal vein. The patient died 6 days after transplantation. Autopsy findings noted fungal organisms morphologically consistent with Mucorales in the liver parenchyma and blood vessels. Tissues were further evaluated at CDC, where mucormycetes were identified by immunohistochemical examination (Figure). Two other recipients received the donor’s right and left kidneys. Neither recipient received antifungal prophylaxis nor had clinical signs or symptoms consistent with an IMI.

**Figure:**
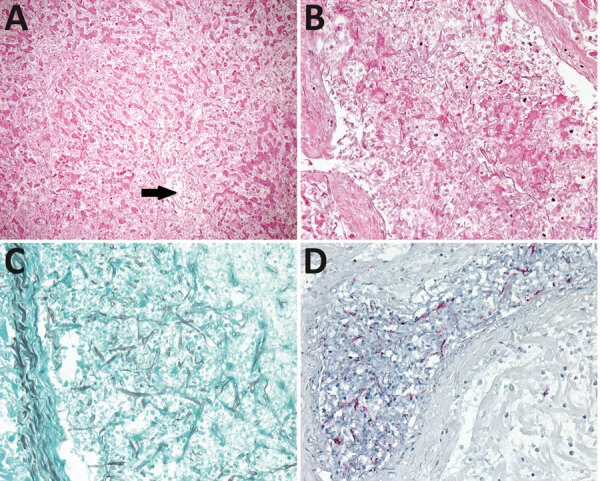
Invasive fungal infection in transplanted liver from an organ donor who drowned, United States (case 2). A) Centrilobular hepatic necrosis without substantial inflammation; fungal hyphae are in a central vein (arrow) and throughout the sinusoids. Hematoxylin–eosin; original magnification ×100. B) Numerous fungal hyphae are in the lumen and wall of a large hepatic vessel. Hematoxylin–eosin; original magnification ×200. C) Grocott methenamine silver stain highlights ribbon-like, pauciseptate, branching funal hyphae within the hepatic vessel. Original magnification ×200. D) Intravascular fungal hyphae stain red by immunohistochemical assay for mucormycete fungi. Original magnification ×200.

For case 3, the donor was a 41-year-old man who fell into a lake after having a seizure. At the hospital, a large quantity of dark fluid was suctioned from his lungs. On bronchoscopic examination, clinicians noted black particulate matter in the lungs; no leukocytes or organisms were seen in the bronchial washings. Organ procurement occurred ≈5 days after hospital admission. Histopathologic examination of lung tissue at CDC showed severe aspiration pneumonia. Culture and polyfungal immunohistochemical assays on donor lung tissue, performed at CDC after illness in a recipient was reported, were negative for fungal pathogens. The recipient of the liver and right kidney (index recipient) was a 66-year-old man with end-stage liver disease and chronic kidney disease who received a diagnosis of scedosporiosis ≈1 month posttransplant and eventually had brain lesions consistent with fungal abscesses, as previously published (*5*). He died 8 weeks after transplantation. Three other patients received the pancreas, heart, and left kidney. After DTAC notified transplant centers of the index case, all 3 recipients were started on voriconazole prophylaxis; none had onset of scedosporiosis.

## Conclusions

We describe 3 investigations of potentially donor-derived IMIs resulting in 4 fatal organ recipient infections, although IMI was not definitively identified in these drowned donors. The 4 recipients tested positive for fungi a median of 7 days (range 3–26 days) and died a median of 8 days (range 4–55 days) after transplantation. All recipients who tested positive died. Onset of disease and outcomes were faster and more severe than reported in previously published literature on donor-derived IMIs, which described a median time of 21 days (range 9 days–6 months) to disease onset and a death rate of 17%; the shortest time to onset of donor-derived mucormycosis described by Gomes and Singh was from a drowned donor (*3*,*6*). These findings highlight the importance of early diagnosis, timely treatment, and prompt communication among transplant centers to facilitate early prophylaxis, diagnosis, and treatment of other recipients when indicated. Transplant teams can also consider antifungal prophylaxis with drugs active against Mucorales and *Scedosporium* species in SOT recipients receiving organs from drowned donors.

One limitation of our study is that these investigations are neither inclusive nor representative of all potential donor-derived IMIs in the United States, given that DTAC relies on passive reporting. Donor drowning circumstances (e.g., type of water and water temperature) likely affect the probability of an IMI after drowning, but this information was not generally available in the donor medical records. Determining whether an IMI was donor-derived is challenging because SOT recipients can be exposed to molds through multiple sources (e.g., healthcare-associated exposures and community exposures) both before and after transplant. Exposed donors may not have detectable pulmonary disease even in the presence of mold; average incubation periods for IMIs in drowning victims are longer than the time between drowning and organ procurement in the described cases (*10*,*11*). Alternatively, angioinvasive molds could have infiltrated from other exposed mucosa or abrasions, leading to dissemination to extrapulmonary organs before procurement. The relatively short times from organ transplantation to symptom onset and the presence of IMIs among multiple recipients of the same donor support a donor origin for infection; previous reports suggest that IMIs in organ transplant recipients have a median time to onset of >180 days (*6*).

Because of the substantial need for organs and considerable mortality rates among persons awaiting transplantation, donors who drowned are considered on a case-by-case basis by transplant clinicians (*12*). Clinicians should maintain clinical suspicion for IMI when caring for SOT recipients receiving organs from drowned donors, communicate concerns about IMIs to other recipient transplant centers in a timely manner, and consider the use of Mucorales- and *Scedosporium*-active antifungal prophylaxis (*13*,*14*).
